# Genomic and functional characterisation of IncX3 plasmids encoding *bla*_SHV-12_ in *Escherichia coli* from human and animal origin

**DOI:** 10.1038/s41598-018-26073-5

**Published:** 2018-05-16

**Authors:** Apostolos Liakopoulos, Jeanet van der Goot, Alex Bossers, Jonathan Betts, Michael S. M. Brouwer, Arie Kant, Hilde Smith, Daniela Ceccarelli, Dik Mevius

**Affiliations:** 1 Department of Bacteriology and Epidemiology, Wageningen Bioveterinary Research, Lelystad, The Netherlands; 2 Department of Infection Biology, Wageningen Bioveterinary Research, Lelystad, The Netherlands; 3 Department of Bacteriology, School of Veterinary Medicine, Faculty of Health and Medical Sciences, University of Surrey, Guildford, United Kingdom; 4 Department of Infectious Diseases and Immunology, Faculty of Veterinary Medicine, Utrecht University, Utrecht, The Netherlands; 5 Present Address: Institute of Biology, University of Leiden, Sylviusweg 72, 2333 BE Leiden, The Netherlands

## Abstract

The *bla*_SHV-12_ β-lactamase gene is one of the most prevalent genes conferring resistance to extended-spectrum β-lactams in Enterobacteriaceae disseminating within and between reservoirs, mostly via plasmid-mediated horizontal gene transfer. Yet, studies regarding the biology of plasmids encoding *bla*_SHV-12_ are very limited. In this study, we revealed the emergence of IncX3 plasmids alongside IncI1α/γ in *bla*_SHV-12_ in animal-related *Escherichia coli* isolates. Four representative *bla*_SHV-12_-encoding IncX3 plasmids were selected for genome sequencing and further genetic and functional characterization. We report here the first complete sequences of IncX3 plasmids of animal origin and show that IncX3 plasmids exhibit remarkable synteny in their backbone, while the major differences lie in their *bla*_SHV-12_-flanking region. Our findings indicate that plasmids of this subgroup are conjugative and highly stable, while they exert no fitness cost on their bacterial host. These favourable features might have contributed to the emergence of IncX3 amongst SHV-12-producing *E. coli* in the Netherlands, highlighting the epidemic potential of these plasmids.

## Introduction

The emerging IncX plasmid family consists of narrow host-range, self-transferable, iteron-containing plasmids with class A theta replication and sizes ranging approximately between 30 and 100 kb^1–3^. IncX plasmids have a highly syntenic backbone; yet based on phylogenetic analysis they can be assigned to six distinct subgroups, namely IncX1 to IncX6^2,4,5^. Although it has been demonstrated that IncX plasmids occurred only infrequently among commensal and pathogenic *E. coli* isolates^6^, plasmids of this family encoding various resistance genes were recently described in Enterobacteriaceae originating from diverse sources and geographical areas^2,4,7–11^. Among this plasmid family, the IncX3 subgroup mediates the spread of genes encoding resistance for clinically relevant first-line (fluoroquinolones and extended-spectrum cephalosporins) and last-resort (carbapenems) antibiotics. IncX3 plasmids have been reported to encode *qnrB*7^9^, *qnrS*1^2,9,12–15^, *bla*_CTX-M-3_^11^, *bla*_SHV-12_^9,16–19^, *bla*_KPC-2_^20,21^, *bla*_KPC-3_^22^, *bla*_NDM-1_^18,19,23^, *bla*_NDM-4_^24^, *bla*_NDM-5_^25-28^, *bla*_NDM-7_^15,26,29-33^, *bla*_NDM-13_^16^, *bla*_NDM-17_^34^ and *bla*_OXA-181_^12–14,35,36^. Overall, these reports highlight the importance of this plasmid subgroup for the dissemination of antibiotic resistance genes within Enterobacteriaceae.

The *bla*_SHV-12_ gene ranks amongst the most predominant extended-spectrum β-lactamases within Enterobacteriaceae of diverse origins^37^. Plasmid-mediated horizontal gene transfer constitutes a key mechanism by which this gene disseminates among bacterial populations, therefore monitoring the spread of plasmids is essential to track the transmission of the *bla*_SHV-12_ gene between different reservoirs^37^. Several plasmid replicon types have been associated with the worldwide dissemination of *bla*_SHV-12_, including A/C, colE, F, HI2, I1α/γ, K, L/M, N, P, R, as well as the recently emerging X3^37^. The few available data on the prevalence of *bla*_SHV-12_-encoding plasmids in the Netherlands, report IncHI2 plasmids in human *Salmonella enterica* isolates, IncN and IncF plasmids in human *Escherichia coli*^37,38^, as well as IncK plasmids in *E. coli* from poultry^37^.

To understand *bla*_SHV-12_ diffusion in the Netherlands, also in light of the emerging role of IncX3 plasmids worldwide, we investigated a collection of previously uncharacterized SHV-12-encoding *E. coli* isolates. We report here the plasmid epidemiology of SHV-12-producing *E. coli* from different reservoirs in The Netherlands, the first fully assembled and annotated sequence of three *bla*_SHV-12_ encoding-IncX3 plasmids of animal origin, and the genetic and functional characteristics of IncX3 plasmids from both human and animal origin.

## Results

### Plasmid epidemiology of *bla*_SHV-12_ and emergence of IncX3

**Figure 1:**
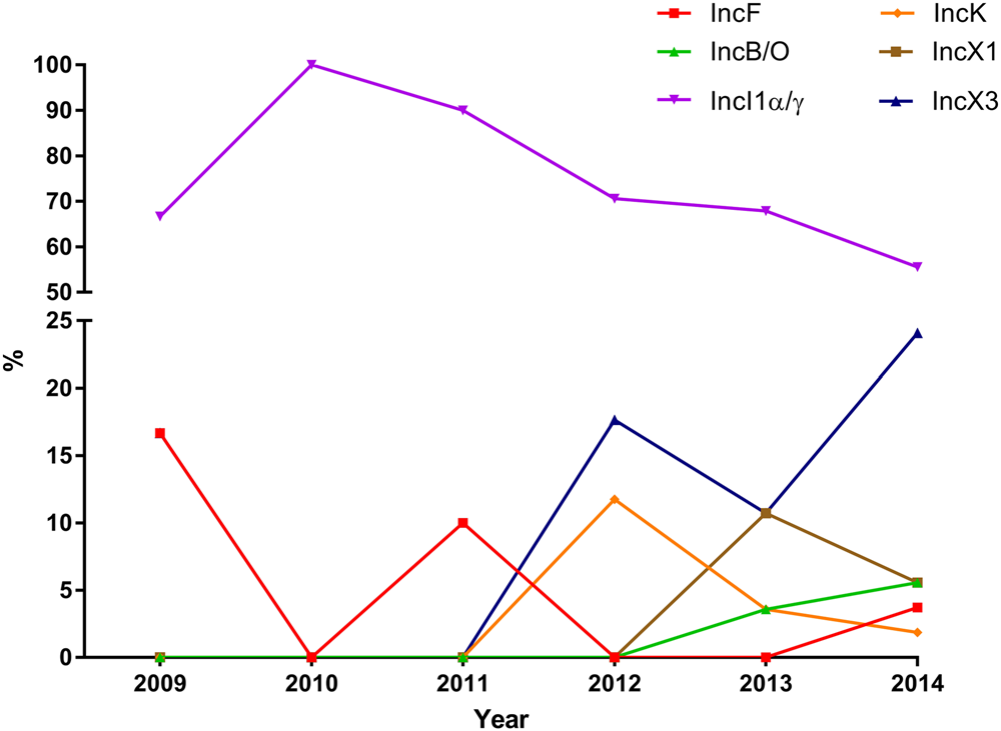
Prevalence of plasmid encoding *bla*_SHV-12_ in *E. coli* isolated between 2009 and 2014 in the Netherlands. Isolates were recovered from food-producing animals, retail meat and humans during national antimicrobial resistance monitoring programmes or national projects. Plasmids belonging to N, colE and F-X1 replicon types were recovered with prevalence of 0.85% and therefore were omitted from the figure.

Among the 129 *bla*_SHV-12_ encoding *E. coli* isolates included in this study (Table 1), 49.6% (n = 64) was isolated from food-producing animals, 41.1% (n = 53) from retail meat and 9.3% (n = 12) from humans. Plasmid typing revealed that *bla*_SHV-12_ was encoded by nine different plasmid families: I1α/γ (n = 86; 66.7%), X3 (n = 21; 16.3%), X1 (n = 6; 4.7%), F (n = 5; 3.9%), B/O (n = 4; 3.1%), K (n = 4; 3.1%), N (n = 1; 0.8%), colE (n = 1; 0.8%) and multi-replicon F-X1 (n = 1; 0.8%). A gradual decrease from 90.0% to 55.6% of IncI1α/γ in parallel with a significant increase from 0.0% to 24.1% (*p* = 0.041) of IncX3 was documented between 2011 and 2014 among food-producing animals and retail meat (Fig. 1). As a result, IncX3 plasmids were among the predominant rep-types encoding *bla*_SHV-12_ in the Netherlands from 2012 onwards (Fig. 1).

**Table 1:** Characteristics of the 129 non-duplicate *bla*_SHV_-encoding *E. coli* isolates included in the study.

Isolate	Date of isolation	Origin	ESBL gene(s)^*^	Inc/rep-type of *bla*_SHV-12_-encoding plasmid
1954014	2009	Livestock (pig)	*bla*_SHV-12_	IncF
35474	2009	Livestock (poultry)	*bla*_SHV-12_	IncF
36289	2009	Livestock (poultry)	*bla*_SHV-12_	IncI1α/γ
36278	2009	Livestock (poultry)	*bla*_SHV-12_	IncI1α/γ
513768	2009	Livestock (poultry)	*bla*_SHV-12_	IncI1α/γ
37318	2009	Livestock (poultry)	*bla*_SHV-12_	IncI1α/γ
35078	2009	Livestock (poultry)	*bla*_SHV-12_	IncI1α/γ
37881	2009	Livestock (poultry)	*bla*_CTX-M-1_ (IncI1α/γ), *bla*_CMY-2_ (IncK), *bla*_SHV-12_	IncX3
35659	2009	Livestock (poultry)	*bla*_CTX-M-1_ (IncN), *bla*_SHV-12_	IncI1α/γ
36498	2010	Livestock (poultry)	*bla*_SHV-12_	IncI1α/γ
36700	2010	Livestock (poultry)	*bla*_SHV-12_	IncI1α/γ
36809	2010	Livestock (poultry)	*bla*_SHV-12_	IncI1α/γ
66191451	2010	Livestock (poultry)	*bla*_SHV-12_	IncI1α/γ
35568	2010	Livestock (poultry)	*bla*_SHV-12_	IncI1α/γ
636942	2010	Livestock (poultry)	*bla*_SHV-12_	IncI1α/γ
1026302	2010	Livestock (poultry)	*bla*_SHV-12_	IncI1α/γ
1025601	2010	Livestock (poultry)	*bla*_SHV-12_	IncI1α/γ
55833907	2011	Livestock (pig)	*bla*_SHV-12_	IncF
55927588	2011	Livestock (pig)	*bla*_SHV-12_	IncI1α/γ
55927758	2011	Livestock (pig)	*bla*_SHV-12_	IncI1α/γ
37156	2011	Livestock (poultry)	*bla*_SHV-12_	IncI1α/γ
36239	2011	Livestock (poultry)	*bla*_SHV-12_	IncI1α/γ
55727422	2011	Livestock (cattle)	*bla*_SHV-12_	IncI1α/γ
884	2011	Livestock (poultry)	*bla*_SHV-12_	IncI1α/γ
1105	2011	Livestock (poultry)	*bla*_SHV-12_	IncI1α/γ
1109	2011	Livestock (poultry)	*bla*_SHV-12_	IncI1α/γ
984	2011	Livestock (poultry)	*bla*_SHV-12_	IncI1α/γ
65268442	2012	Livestock (pig)	*bla*_SHV-12_	IncI1α/γ
36788	2012	Livestock (poultry)	*bla*_SHV-12_	IncI1α/γ
29062012-02	2012	Livestock (poultry)	*bla*_SHV-12_	IncI1α/γ
55580200139	2012	Livestock (poultry)	*bla*_SHV-12_, *bla*_TEM-52c_	IncK
65094754	2012	Livestock (cattle)	*bla*_SHV-12_	IncI1α/γ
36458	2013	Livestock (poultry)	*bla*_SHV-12_	IncI1α/γ
35658	2013	Livestock (poultry)	*bla*_SHV-12_	IncI1α/γ
1399001	2013	Livestock (poultry)	*bla*_SHV-12_	IncX3
859	2014	Livestock (poultry)	*bla*_SHV-12_	IncX3
219	2014	Livestock (poultry)	*bla*_SHV-12_	IncX3
1041	2014	Livestock (poultry)	*bla*_SHV-12_	IncI1α/γ
900	2014	Livestock (poultry)	*bla*_SHV-12_	IncI1α/γ
570	2014	Livestock (poultry)	*bla*_SHV-12_	IncX3
500	2014	Livestock (poultry)	*bla*_SHV-12_	IncB/O
374	2014	Livestock (poultry)	*bla*_SHV-12_	IncI1α/γ
287	2014	Livestock (poultry)	*bla*_SHV-12_	IncX3
**73** ^¥^	2014	Livestock (poultry)	*bla*_SHV-12_	IncX3
1424	2014	Livestock (poultry)	*bla*_SHV-12_	IncX3
71	2014	Livestock (poultry)	*bla*_SHV-12_	IncI1α/γ
828	2014	Livestock (poultry)	*bla*_SHV-12_	IncX1
1003	2014	Livestock (poultry)	*bla*_SHV-12_	IncI1α/γ
11	2014	Livestock (poultry)	*bla*_SHV-12_	IncI1α/γ
240	2014	Livestock (poultry)	*bla*_SHV-12_	IncF-X1
**386** ^¥^	2014	Livestock (poultry)	*bla*_SHV-12_	IncX3
990	2014	Livestock (poultry)	*bla*_SHV-12_	IncI1α/γ
1341	2014	Livestock (poultry)	*bla*_SHV-12_	IncI1α/γ
1420	2014	Livestock (poultry)	*bla*_SHV-12_	IncX3
118	2014	Livestock (poultry)	*bla*_SHV-12_	IncI1α/γ
139	2014	Livestock (poultry)	*bla*_SHV-12_	IncX3
864	2014	Livestock (poultry)	*bla*_SHV-12_	IncX1
1206	2014	Livestock (poultry)	*bla*_SHV-12_	IncI1α/γ
876	2014	Livestock (poultry)	*bla*_SHV-12_	IncI1α/γ
20	2014	Livestock (poultry)	*bla*_SHV-12_	IncX1
229	2014	Livestock (poultry)	*bla*_SHV-12_	IncX3
1096	2014	Livestock (poultry)	*bla*_SHV-12_	IncF
1433	2014	Livestock (pig)	*bla*_SHV-12_	IncX3
1116	2014	Livestock (cattle)	*bla*_SHV-12_	IncI1α/γ
69438407	2012	Meat (poultry)	*bla*_CTX-M-1_ (IncI1α/γ), *bla*_SHV-12_	IncX3
69606962	2012	Meat (poultry)	*bla*_SHV-12_	IncX3
76495084	2012	Meat (beef)	*bla*_SHV-12_	IncI1α/γ
76495084 02	2012	Meat (beef)	*bla*_SHV-12_	IncI1α/γ
69843204	2012	Meat (poultry)	*bla*_SHV-12_	IncI1α/γ
69210023	2012	Meat (poultry)	*bla*_SHV-12_	IncK
69843409	2012	Meat (poultry)	*bla*_SHV-12_	IncI1α/γ
69438105	2012	Meat (poultry)	*bla*_SHV-12_	IncX3
69585604	2012	Meat (poultry)	*bla*_SHV-12_	IncI1α/γ
69064655	2012	Meat (poultry)	*bla*_SHV-12_	IncI1α/γ
69770576	2012	Meat (poultry)	*bla*_SHV-12_, *bla*_TEM-52c_	IncI1α/γ
69477895	2012	Meat (poultry)	*bla*_SHV-12_, *bla*_TEM-52c_	IncI1α/γ
69927807	2013	Meat (poultry)	*bla*_CMY-2_ (IncK), *bla*_SHV-12_	IncB/O
698975250004	2013	Meat (poultry)	*bla*_SHV-12_	colE
699561810004	2013	Meat (poultry)	*bla*_SHV-12_	IncI1α/γ
699819760004	2013	Meat (poultry)	*bla*_SHV-12_	IncI1α/γ
69986056	2013	Meat (poultry)	*bla*_SHV-12_	IncI1α/γ
693784120004	2013	Meat (poultry)	*bla*_SHV-12_	IncI1α/γ
693785440004	2013	Meat (poultry)	*bla*_SHV-12_	IncI1α/γ
693562060004	2013	Meat (poultry)	*bla*_SHV-12_	IncI1α/γ
699898610004	2013	Meat (poultry)	*bla*_SHV-12_	IncI1α/γ
699229530004	2013	Meat (poultry)	*bla*_SHV-12_	IncI1α/γ
698980410004	2013	Meat (poultry)	*bla*_SHV-12_	IncI1α/γ
69960219	2013	Meat (poultry)	*bla*_SHV-12_	IncI1α/γ
69960316	2013	Meat (poultry)	*bla*_SHV-12_	IncI1α/γ
69960316	2013	Meat (poultry)	*bla*_SHV-12_	IncI1α/γ
69345093	2013	Meat (pork)	*bla*_SHV-12_	IncI1α/γ
69799639	2013	Meat (beef)	*bla*_SHV-12_	IncI1α/γ
699799710004	2013	Meat (beef)	*bla*_SHV-12_	IncI1α/γ
694658380004	2013	Meat (poultry)	*bla*_SHV-12_, *bla*_SHV-2A_, *bla*_TEM-52c_ (IncI1α/γ)	IncI1α/γ
699813050004	2013	Meat (poultry)	*bla*_SHV-12_, *bla*T_EM-52c_ (IncI1α/γ)	IncI1α/γ
699900020004	2013	Meat (poultry)	*bla*_SHV-12_	IncK
693503480004	2013	Meat (poultry)	*bla*_SHV-12_	IncX1
699953490004	2013	Meat (poultry)	*bla*_SHV-12_	IncX1
699081950004	2013	Meat (poultry)	*bla*_SHV-12_	IncX1
699952170004	2013	Meat (poultry)	*bla*_SHV-12_	IncX3
**69960189** ^¥^	2013	Meat (poultry)	*bla*_SHV-12_	IncX3
79158224	2014	Meat (beef)	*bla*_SHV-12_	IncF
M14P0112	2014	Meat (poultry)	*bla*_SHV-12_	IncI1α/γ
79197637	2014	Meat (poultry)	*bla*_SHV-12_	IncI1α/γ
79059943	2014	Meat (poultry)	*bla*_SHV-12_	IncI1α/γ
79230383	2014	Meat (poultry)	*bla*_SHV-12_	IncB/O
79696536	2014	Meat (poultry)	*bla*_SHV-12_	IncI1α/γ
79194778	2014	Meat (pork)	*bla*_SHV-12_	IncI1α/γ
79195006	2014	Meat (poultry)	*bla*_SHV-12_	IncI1α/γ
79156655	2014	Meat (poultry)	*bla*_SHV-12_	IncI1α/γ
79207004	2014	Meat (poultry)	*bla*_SHV-12_	IncI1α/γ
79626295	2014	Meat (poultry)	*bla*_SHV-12_	IncB/O
79207101	2014	Meat (poultry)	*bla*_SHV-12_	IncI1α/γ
79352292	2014	Meat (poultry)	*bla*_SHV-12_	IncK
79771872	2014	Meat (poultry)	*bla*_SHV-12_	IncX3
79445126	2014	Meat (poultry)	*bla*_SHV-12_	IncI1α/γ
79042587	2014	Meat (poultry)	*bla*_SHV-12_	IncX3
**1190900169** ^¥^	2009	Human (urine)	*bla*_SHV-12_	IncX3
1190900881	2009	Human (urine)	*bla*_SHV-12_	IncI1α/γ
1190900890	2009	Human (urine)	*bla*_SHV-12_	IncI1α/γ
306	2014	Human (faeces)	*bla*_SHV-12_	IncI1α/γ
1.1	2014	Human (faeces)	*bla*_SHV-12_	IncI1α/γ
1.58	2014	Human (faeces)	*bla*_SHV-12_	IncI1α/γ
2.12	2014	Human (faeces)	*bla*_SHV-12_	IncI1α/γ
2.25	2014	Human (faeces)	*bla*_SHV-12_	IncI1α/γ
2.48	2014	Human (faeces)	*bla*_SHV-12_	IncI1α/γ
2.49	2014	Human (faeces)	*bla*_SHV-12_	IncI1α/γ
2.52	2014	Human (faeces)	*bla*_SHV-12_	IncI1α/γ
1.71	2014	Human (faeces)	*bla*_SHV-12_	IncN

^*^When known the inc/rep-type of the plasmid encoding ESBL genes (excluding the *bla*_SHV-12_) is given in parenthesis.

^¥^In bold the 4 plasmids sequenced and functionally characterized.

To further study these emerging IncX3 plasmids, four of them (pEC-NRS18, pEC-393, pEC-125 and pEC-243) randomly selected from human-related *E. coli* ST69 and from diverse *E. coli* STs of animal origin (ST117, ST315 and ST410) were fully sequenced and functionally characterized in this study (Table 2).

**Table 2:** IncX3 plasmids included in this study and their characteristics.

Plasmid ID	Year	Host	Host source	Resistance gene(s)	Size (bp)	GC Content %	Open reading frames
pEC-NRS18	2009	*E. coli* ST69/CC69	Human UTI*	*bla*_SHV-12_, *bla*_TEM-1_, *qnrS*1	48,250	46.4	74
pEC-393	2013	*E. coli* ST410/CC23	Turkey meat	*bla*_SHV-12_	43,506	46.8	65
pEC-125	2014	*E. coli* ST117	Chicken faeces	*bla*_SHV-12_, *qnrS*1	46,338	46.4	73
pEC-243	2014	*E. coli* ST315/CC38	Chicken faeces	*bla*_SHV-12_, *qnrS*1	46,338	46.4	73

^*^Urinary tract infection.

### IncX3 plasmid backbone is highly syntenic and conserved

**Figure 2:**
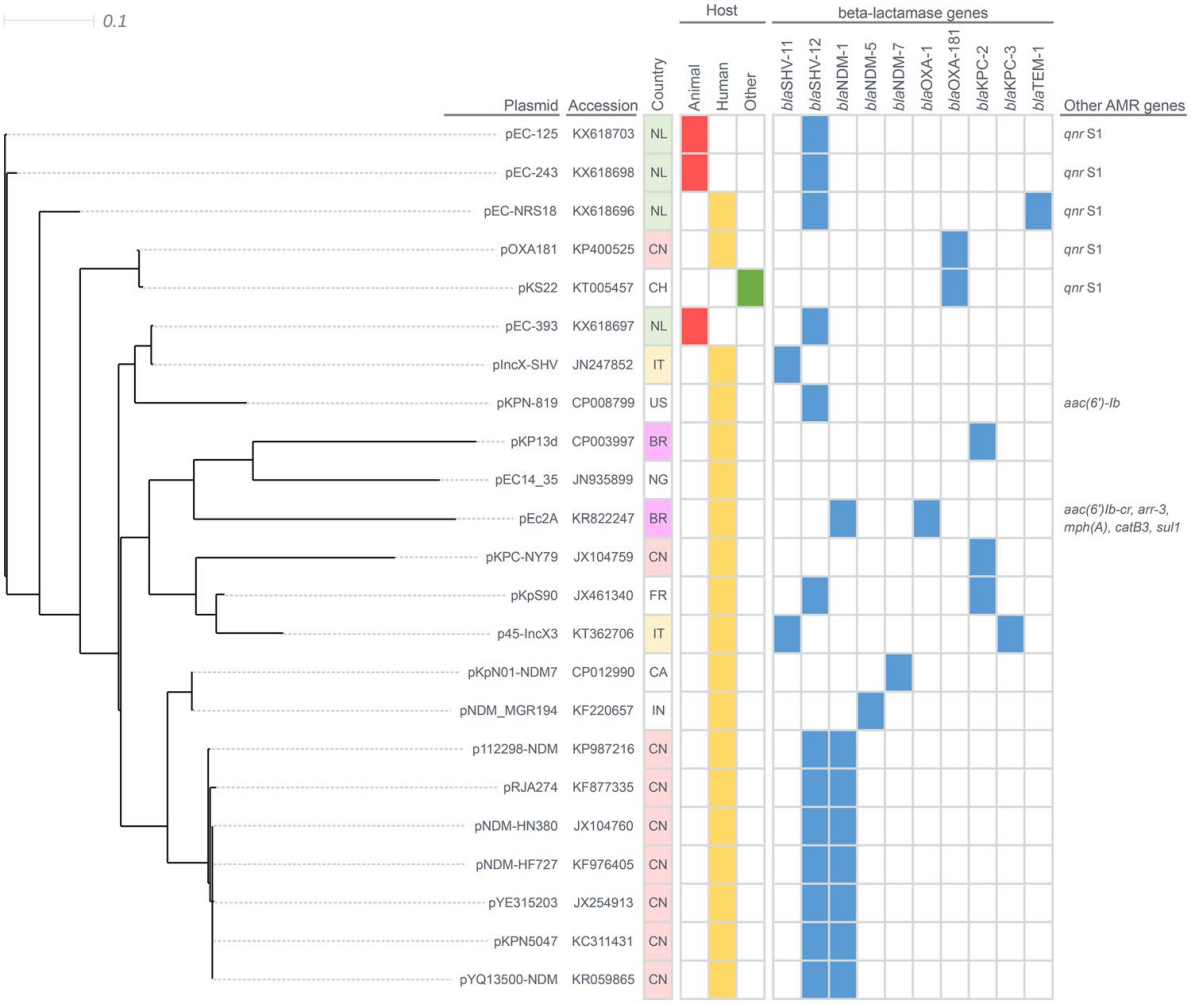
BioNJ MUMi distances phylogram of IncX3 plasmids. Plasmid sequences obtained here and those available in GenBank database were compared pair-wise and maximum unique matches converted to MUMi distances were hierarchically clustered and displayed as a phylogram using the BioNJ algorithm. GenBank accession number, antibiotic resistant gene content, country and source of isolation are indicated. NL: Netherlands, CN: China, CH: Switzerland, IT: Italy, US: United States, BR: Brazil, NG: Nigeria, FR: France, CA: Canada, IN: India and AMR: antimicrobial resistance.

Comparison between whole sequences of the four IncX3 plasmids from this study and twenty IncX3 plasmids available in GenBank revealed a highly conserved plasmid backbone and their organization into a number of distinct clades (Fig. 2). Plasmids of animal origin pEC-125 and pEC-243 were closely clustered together (MUMi distance 0.018) and grouped with the human-derived plasmid pEC-NRS18 that showed distance from 0.085 (pEC-125) to 0.093 (pEC-243). The animal-derived plasmid pEC-393 (turkey meat) clustered with a *Klebsiella pneumoniae*-encoded pIncX-SHV from human source in Italy (MUMi distance 0.002). IncX3 plasmids recovered in the Netherlands clustered closely with pOXA181 (China) and pKS22 (Switzerland) encoding *bla*_OXA-181_ and *qnrS*1, respectively, with MUMi distances varying from 0.140 (pOXA181 with pEC-125) to 0.179 (pKS22with pEC-393).

**Figure 3:**
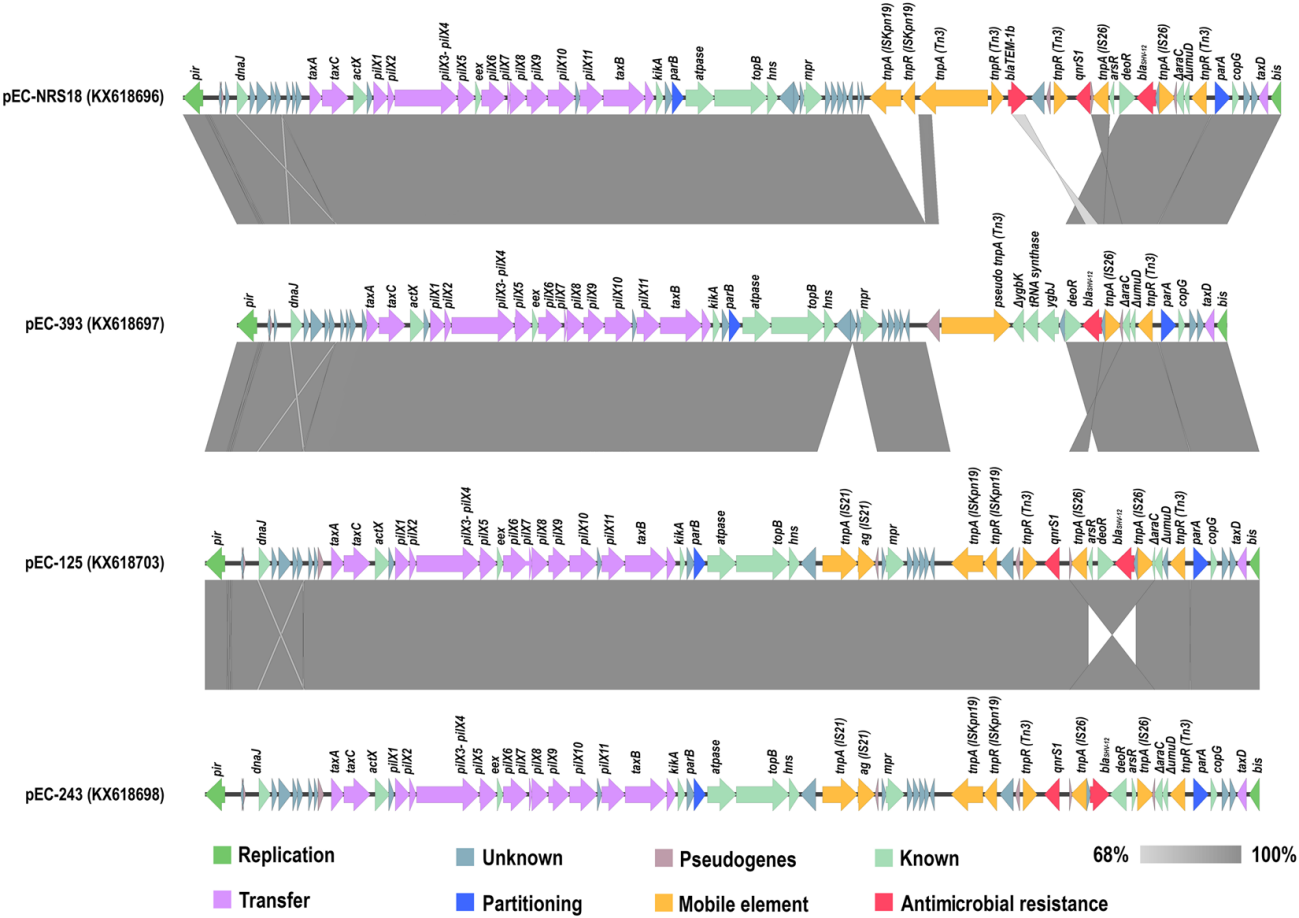
Linear comparison in scale of IncX3 plasmids. The open reading frames identified in each sequence are represented with arrows, with the arrowhead indicating the direction of transcription. Their involvement in replication, partitioning, transfer, or antibiotic resistance, their association to mobile genetic elements, as well as other known or unknown functions and pseudogenes are colour-coded. Areas shaded in grey indicate nucleotide identity.

The four plasmids sequenced, assembled and fully annotated in this study had sizes varying from 43,506 (pEC-393) to 48,250 (pEC-NRS18) bp with an average GC content of 46.5% (Table 2). Similar to other IncX3 plasmids, they carried three putative origins of replication (*oriV-α*, *oriV-β* and *oriV-γ*), two origins of transfer (*oriT-α* and *oriT-β*), and approximately 6 iteron sequences. Nucleotide sequence analysis revealed 65 to 74 predicted open reading frames (Fig. 3). Comparative genomic of all four plasmids (Fig. 3) revealed high synteny among them, encoding genes for replication (*pir*: replication initiation protein and *bis*: replication accessory protein), partitioning (*parAB*), entry exclusion (*eex*), maintenance (*topB* and *hns*), transcriptional activation (*actX*), and conjugative transfer [*pilX1-11* (type IV secretion system) and *taxA-C*]. In addition, a mosaic variable region containing resistance genes as well as intact and/or defective insertion sequences (i.e. *IS*21, *ISKpn*19, Tn3, and *IS*26) was identified in all four plasmids upstream of the partitioning gene *parA* (Fig. 3).

The variable region of pEC-NRS18 contained *bla*_TEM-1_ embedded in a Tn3 transposon, as well as genes *bla*_SHV-12_ and *qnrS*1 associated with the upstream presence of *IS*26 in the opposite and same orientation, respectively. Similarly, pEC-125 and pEC-243 contained both *bla*_SHV-12_ and *qnrS*1 genes, whereas pEC-393 encoded only *bla*_SHV-12_ associated to *IS*26. The genetic environment surrounding *bla*_SHV-12_ was characterized by two flanking copies of *IS*26 distributed in opposite orientation to form a composite *IS*26-*IS*26 transposon (3,633 bp); this structure was conserved in three of the four IncX3 plasmids (pEC-NRS18, pEC-125 and pEC-243; Fig. 3). BLAST analysis revealed that this composite transposon is 100% identical to previously described transposons located on plasmids of *K. pneumoniae* (IncFIB; GenBank accession no. CP019048.1) and *Aeromonas veronii* (IncA/C2; GenBank accession no. CP014775.1), as well as into the genome of *Pseudomonas aeruginosa* (GenBank accession no. GU592828.1). The flanking region of *bla*_SHV-12_ on pEC-393 showed a partial 2,484-bp overlap with corresponding regions of pEC-NRS18, pEC-125 and pEC-243 (Fig. 3) and encoded genes *deoR*, *ygbJ* and truncated *ygbK* also present on several *K. pneumoniae* chromosomes (GenBank accession no. CP000647, CP002910 and CP008831). This 4,783-bp region exhibits 100% identity to a fragment of a *bla*_SHV_-harboring IncR plasmid of *K. pneumoniae* (GenBank accession no. KF954150.1).

### IncX3 plasmid transfer is temperature-dependent

**Figure 4:**
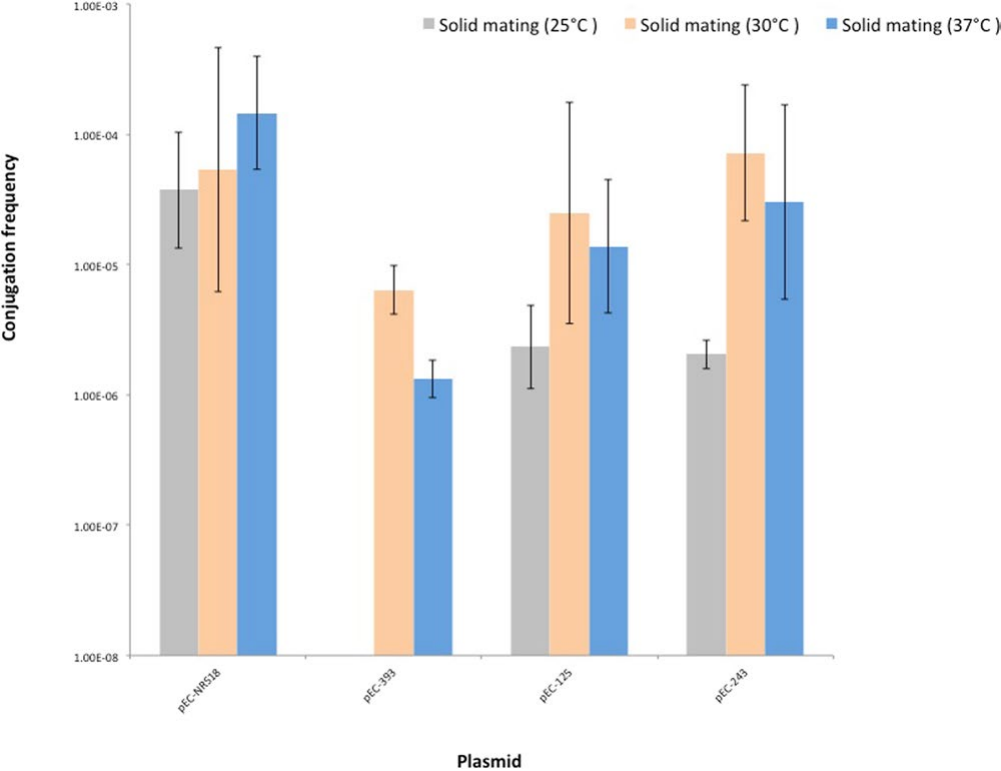
Conjugation frequencies of IncX3 plasmids. The reported values represent the average of three independent solid mating experiments (25 °C, 30 °C and 37 °C) and the error bars the standard deviation.

Conjugation frequencies of the four IncX3 plasmids was determined and results are shown in Fig. 4. Transfer rates differed between solid matings at different temperatures. Geometric mean frequencies ranged below the detection limit (≤1 × 10^−9^, pEC-393) to 3.73 × 10^−5^ (pEC-NRS18) at 25 °C, from 6.36 × 10^−6^ (pEC-393) to 7.16 × 10^−5^ (pEC-243) at 30 °C, and from 1.33 × 10^−6^ (pEC-393) to 1.46 × 10^−4^ (pEC-NRS18) at 37 °C. The analysis showed a significant difference between conjugation frequencies at different temperatures (*p* = 0.027), mainly due to lower frequencies at 25 °C compared with frequencies at 30 °C. The difference in conjugation frequencies between different plasmids was nearly significant (*p* = 0.054). Comparisons of single plasmids at 30 °C and 37 °C indicated differences between the plasmids at 37 °C, with lower conjugation frequencies for animal-derived plasmids (pEC-125, pEC-243 and pEC-393), and higher frequencies for the human-derived plasmid pEC-NRS18. Overall, plasmid of animal origins seem to transfer better at 30 C, conversely to the human-derived plasmid (Fig. 4).

### IncX3 plasmids exert no fitness cost to the bacterial host and are highly stable

**Figure 5:**
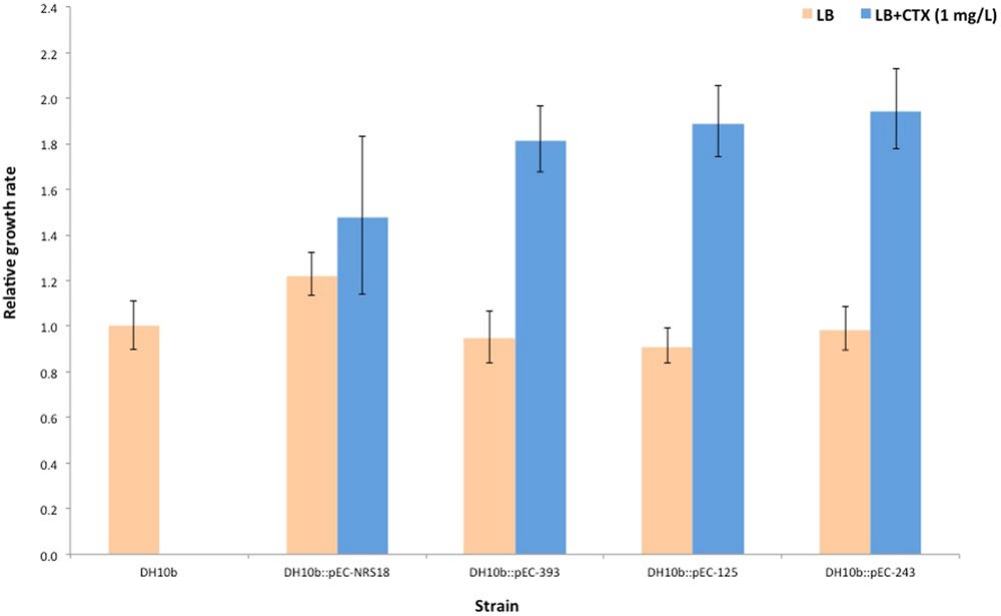
Relative exponential growth rates of IncX3-harbouring plasmid *E. coli* DH10b strains. All growth rates are set relative to plasmid-free *E. coli* DH10b. The reported values represent the average of three independent experiments and the error bars represent the 95% confidence interval for the ratio.

The cost of IncX3 plasmid presence on the host cell fitness was assessed in the absence and in the presence of cefotaxime by comparing the exponential growth rate of *E. coli* DH10b with and without plasmid (Fig. 5). The exponential growth rates of *E. coli* DH10b harbouring each of the IncX3 plasmids singularly varied from 0.91 (95% CI 0.84–0.99; DH10b::pEC-125) to 1.22 (95% CI 1.13–1.32; DH10b::pEC-NRS18) in the absence of antibiotic selective pressure, and from 1.48 (95% CI 1.14–1.83; DH10b::pEC-NRS18) to 1.94 (95% CI 1.77–2.12; DH10b::pEC-243) in the presence of cefotaxime. In the absence of cefotaxime, DH10b::pEC-125 showed significantly lower (*p* = 0.04) and DH10b::pEC-NRS18 significantly higher (*p* < 0.001) exponential growth rates compared to *E. coli* DH10b control strain. Pairwise comparison of the relative growth rates for *E. coli* DH10b harbouring each of the IncX3 plasmids in the absence and presence of cefotaxime indicated significantly higher rates in the presence of selective pressure (*p* < 0.001) except for DH10b::pEC-NRS18.

*E. coli* DH10b transformed strains were also propagated without positive antibiotic selective pressure as a measure of plasmid stability. After approximately 180 generations of growth, the percentage of plasmid-harbouring cells in each population (for all four plasmids singularly) was determined. All plasmids were found to be stably maintained in the *E. coli* population, ranging from 99.9% (95% CI 99.98–99.99) (pEC-NRS18) to 100% (95% CI 99.9–100) (pEC-393, pEC-125 and pEC-243) plasmid-harbouring cells per generation.

### IncX3 plasmids do not contribute to bacterial pathogenicity

**Figure 6:**
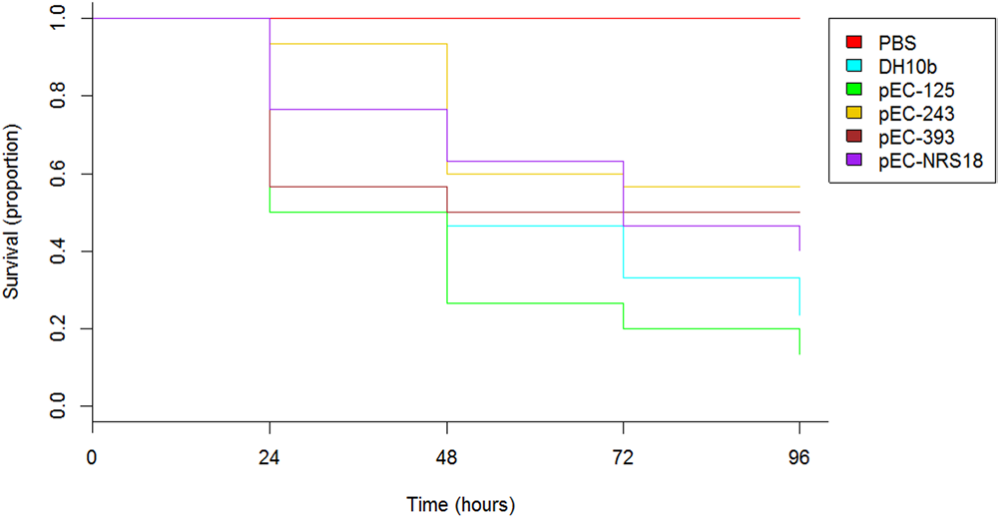
Impact of harbouring an IncX3 plasmid on *E. coli* DH10b strain pathogenicity. Kaplan-Meier plot of *G. mellonella* survival after injection with 10^7^ CFU/larva of plasmid-free and IncX3-harbouring plasmid (pEC-NRS18, pEC-393, pEC-125 and pEC-243) *E. coli* DH10b strain is shown. Experiments were performed in triplicate and the plot represents the combined (additive) data from all experiments. No larval death was observed in control larvae injected with an equivalent volume of PBS.

Annotation of the four IncX3 plasmids revealed the presence of a Type 4 Secretion System (*pilX1-11*) and several ORFs with unknown function that could potentially act as virulence effectors (Fig. 3). The *Galleria mellonella in vivo* infection model was employed to evaluate the impact of harbouring an IncX3 plasmid on bacterial pathogenicity. The LD_50_ value after 24 h was determined to be 10^7^ CFU/larvae and survival curves were compared between the isogenic control *E. coli* DH10b strain and DH10b transformed strains harbouring each of the IncX3 plasmids (Fig. 6). All four transformed strains carrying IncX3 plasmids displayed comparable virulence to the plasmid-free control strain (mortality = 40–86%), with no significant difference in the 96 h survival curves (Fig. 6). In both control groups all larvae survived.

## Discussion

Our results confirmed that IncI1α/γ plasmids are the major facilitators of the *bla*_SHV-12_ diffusion in *E. coli* of human and animal origin and mirrored the global plasmid repertoire associated with *bla*_SHV-12_^37,39^. However, a gradual decrease in the prevalence of IncI1α/γ and a parallel increase in IncX3 plasmids encoding *bla*_SHV-12_ was documented mostly in animal-related commensal *E. coli*. Previously, the IncX3 plasmid subgroup was only incidentally associated with *bla*_SHV-12_^9,16–19^ and/or *qnrS*1^2,9,12–15^ among clinically recovered *E. coli* isolates, and very recently it was identified among poultry isolates in Germany^40^. IncX3 plasmids have been documented in other Enterobacteriaceae worldwide in association with multi-resistance, including to carbapenems^18,20,22,24,26,34,36^. Nevertheless, no association between IncX3 plasmids and other resistance genes (apart from *bla*_SHV-12,_ *bla*_TEM-1_ and *qnrS*1) in Enterobacteriaceae was found in the Netherlands (data not shown).

The high degree of synteny and conservation in the backbone of IncX3 plasmids among *E. coli* isolates of both human and animal origin reflects the ecological success of this plasmid subgroup^2^. In addition to encoding genes essential for their maintenance and dissemination, IncX3 plasmids contained a variable region encoding resistance to clinically important antimicrobial agents (fluoroquinolones and/or extended spectrum cephalosporins). Our findings confirm the potential of this subgroup for accumulation of resistance genes via IS-mediated transposition, with the likely consequence of limiting effective treatment options for possible human infections^9,13,15,16,18,21^. As previously described^41^, the presence of *IS*26 linked to *bla*_SHV-12_ and other co-linear genes originating from the chromosome of *K. pneumoniae* was documented within the IncX3 variable region, confirming the hypothesis of *IS*26-mediated mobilization of a *bla*_SHV_ ancestor gene from the chromosome of *K. pneumoniae*^42^. Although we cannot prove how *bla*_SHV-12_ was integrated into IncX3 plasmids, the documented ability of *IS*26 to participate in both replicative transposition and self-targeted transposition creating *IS*26-bounded transposons^43^ could have facilitated the formation of the composite *bla*_SHV-12_-encoding *IS*26-*IS*26 transposon seen in the majority of the IncX3 plasmids studied here. The presence of identical composite transposons, mostly on plasmids of diverse replicon types, indicates its mobilization ability preferentially onto plasmids rather than the chromosome, as previously suggested^44^. In addition to the contribution of *IS*26 to the mobilization on conjugative plasmids and the subsequent dissemination of *bla*_SHV-12_, it has been shown that *IS*26 supplies a promoter −35 box that can be coupled with a −10 box in the adjacent DNA^45^, possibly also contributing to the expression of this resistance gene.

IncX3 plasmids, as well as the archetypal R6K plasmid of the IncX family, have been investigated for their ability to conjugate^13,15,16,18,19,46^. We documented the temperature-dependent transfer of animal-derived IncX3 plasmids with frequencies higher at 30 °C, suggesting a more efficient transfer in the environment than in the animal gut. Similarly to other conjugative plasmids, IncX3 plasmids hold a gene encoding a H-NS-like protein^2,47–51^. Several studies demonstrated the inhibitory role of H-NS-like proteins on gene thermoregulation owing to their ability to polymerize along and bridge adjacent DNA regions at 37 °C and to derepress H-NS-regulated genes at lower temperatures^47,48,52^. The involvement of these proteins in the temperature-dependent conjugative transfer of IncHI1 plasmids suggests a similar role for IncX3 plasmids that can only be speculated here. The ability of IncX3 plasmids to replicate and be stably maintained in α-, β- and γ-Proteobacteria^53^, in combination with a higher conjugation frequency at 30 °C, underscore a potential mesophilic Proteobacteria reservoir of this plasmid subgroup.

In contrast with studies describing that plasmids impose fitness cost on their bacterial hosts^54^, growth kinetics obtained over 24 h showed no evidence of fitness cost on the bacterial *E. coli* host. It has been demonstrated that H-NS-like proteins are able to silence newly introduced foreign sequences (including plasmids), based on increased adenine and thymine (AT) content in comparison with the chromosome^51,55,56^. Taking into consideration the high AT content of the IncX3 plasmids, we hypothesize that the *H-NS* gene present on these plasmids allows them to invade bacterial hosts with a minimal impact on their fitness, ensuring the future competitiveness of the new plasmid-host combination even without the presence of antibiotic selective pressure. The significant IncX3 plasmid-mediated fitness enhancement of *E. coli* under antibiotic selective pressure highlights the ecological advantage and subsequent successful proliferation of these plasmids in antibiotic-rich reservoirs.

All IncX3 plasmids encoded the widespread partitioning system ParAB ensuring the correct inheritance of these plasmids to the daughter cells^57^. The observed high stability of IncX3 plasmids is potentially due to their high conjugation frequency and absence of a fitness burden, as well as to a low rate of segregational loss.

Our data show that IncX3 plasmids encode a type IV secretion system (T4SS, *pilX1-11*), typically used for the exchange of genetic material within bacteria, toxin secretion and the translocation of virulent effector proteins into eukaryotic host cells^58,59^. Yet, a virulence potential of IncX3-carrying *E. coli* DH10b was not observed, suggesting that T4SS does not play a role in the virulence of *E. coli*, at least in the *G. mellonella* model, conversely from other Gram-negative pathogens^58^.

In conclusion, we report the first genetic characterisation of IncX3 plasmids of animal origin, as well as the first functional analysis of human- and animal-derived plasmids of this subgroup, including their conjugation frequencies, stability, fitness cost and virulence potential. IncX3 plasmids are highly conserved, syntenic, conjugative and highly stable, while they exert no fitness cost on their bacterial host, independent of their origin. Although clonal expansion of *E. coli* strains could also play a role as suggested by our finding in The Netherlands of *E. coli* from the same clonal complex and carrying the same IncX3 plasmid (data not shown), the favourable plasmid functional features potentially contributed to their emergence amongst SHV-12-producing *E. coli* in the Netherlands, highlighting the epidemic potential of this plasmid subgroup.

## Materials and Methods

### Bacterial strains, transformants and plasmids

A total of 129 non duplicate *bla*_SHV-12_ encoding *E. coli*, consecutively recovered during national antimicrobial resistance monitoring programmes or national projects between 2009 and 2014 were included in the study^60^. Identification of the isolates was performed by MALDI-TOF Mass spectrometry (Brucker, Coventry, UK). Genes conferring an ESC^R^ phenotype were sought by microarray analysis followed by PCR amplification and sequencing^61^. Plasmid location of *bla*_SHV-12_ was determined using a transformation-based approach. Briefly, plasmids encoding *bla*_SHV-12_ were extracted from the parental strain using a miniprep method and transformed into *E. coli* DH10b cells (Invitrogen, Van Allen Way, CA USA) by electroporation under the following conditions: 1.25 kV/cm, 200 Ω, 25 μFar, as previously described^61^. Transformants were selected on Luria-Bertani (LB) agar plates supplemented with cefotaxime (1 mg/L) and confirmed for the presence of *bla*_SHV-12_ gene. The presence of a single plasmid in the transformants was confirmed by S1-PFGE on the transformants, followed by Southern blot hybridization using DIG-labelled probe (DIG DNA Labeling and Detection Kit, Roche, Mannheim, Germany) targeting the *bla*_SHV-12_ gene, as previously described^62^. Plasmid typing was confirmed by PCR-based replicon typing (PBRT KIT, DIATHEVA, Fano, Italy) on the transformants. Host *E. coli* sequence type were assigned by MLST based on the allelic profiles of seven housekeeping genes (*adk*, *fumC*, *gyrB*, *icd*, *mdh*, *purA* and *recA*)^63^.

### Plasmid sequencing, assembly and analysis

Four genetically and epidemiologically unrelated IncX3 plasmids encoding *bla*_SHV-12_ were randomly selected for further analysis from *E. coli* isolates belonging to the single ST of human origin (ST69) and diverse STs of animal origin, including the predominant animal-related ST (ST117). The relevant characteristics of the selected plasmids are specified in Table 2. Plasmid DNA from transformants was isolated using the QIAfilter Plasmid Midi Kit (QIAGEN, Hilden, Germany) according to the manufacturer’s recommendations. Deep sequencing of the plasmid genomes was performed using 300-bp paired-end sequencing libraries (Nextera TAG-mentation sequencing kits [Epicentre]) on an Illumina MiSeq sequencer. High-quality filtered reads were subsequently assembled *de novo* using SPAdes algorithm (SPAdes version 3.7.1) for Illumina-derived reads and then manually curated to close the gaps. Putative open reading frames (ORFs) were identified by RAST version 2.0 and manually curated when necessary^64^. BLASTP analyses of the putative ORFs against the NCBI non-redundant proteins (NR) database, Pfam, and Interpro scan were used to assess their putative functions by identification of structural features and motifs^65,66^. ResFinder (version 2.1), PlasmidFinder (version 1.3) and ISfinder were used to determine the presence of resistance genes, replicon types and insertion sequences, respectively^67–69^. Plasmid sequences were hierarchically clustered and displayed as a phenogram using the BioNJ algorithm, where the underlying distance matrix was calculated from the pairwise non-overlapping maximal unique matches (MUMs) using Nucmer version 3.07^70,71^. Relative pairwise distances were obtained by dividing the pairwise MUMs’ sum by the average genome size of the two paired genomes (MUMi genomic distance)^72^. BioNJ trees were generated from the MUMi distance matrix using SplitsTree4^73^. BLAST analysis was used to assess sequence identity between the *bla*_SHV-12_-surrounding region and nucleotide sequences deposited to NCBI^74^.

### Mating assays

Plasmid conjugation was assessed in solid mating assays at 25 °C, 30 °C and 37 °C conducted in triplicate. Chloramphenicol resistant (chlor^R^) *E. coli* MG1655::*yfp* was used as a recipient strain in 1:1 ratio with donor *E. coli* DH10b transformed strains carrying the different IncX3 plasmids, as previously described^75^. Overnight cultures of recipient and donor strains in mid-exponential phase were co-incubated (100 μl each) onto sterile nitrocellulose filters of 0.45 μm pore size (Schleicher and Schuell GmbH, Dassel, Germany) for 4 h at 25 °C, 30 °C and 37 °C. Transconjugants were selected on LB agar supplemented with chloramphenicol (32 mg/L) and cefotaxime (1 mg/L). Positive transconjugants were confirmed by PCR amplification for the resistance and *yfp* genes. Conjugation frequency was calculated as the number of transconjugants per donor cell, with the absence of transconjugants suggesting either non-conjugative plasmids or conjugation frequencies below the detection limit (≤1 × 10^−9^). For statistical analysis, conjugation frequencies were transformed to log10 values, the differences between the temperatures and plasmids were tested using a non-parametric Kruskal-Wallis test, and a *p* value < 0.05 was considered to be statistically significant. All analysis was performed using R and RStudio (version 1.0.143)^76,77^.

### Fitness cost assays

Liquid cultures of *E. coli* DH10b transformed strains carrying different IncX3 plasmids were incubated overnight in 3 mL LB medium at 37 °C with 180 rpm shaking. Cultures were then diluted 100-fold into 3 mL of fresh pre-warmed LB medium with and without antibiotic (1 mg/L of cefotaxime) and incubated under the same conditions until mid-exponential phase (OD_600_ of ≈0.5). 200 µL of each culture were loaded in triplicate in wells of a 100-well honeycomb plate and incubated at 37 °C with shaking for 24 h. Growth rates were obtained by measuring optical density at 600 nm every 30 min by using a Bioscreen C Reader (Oy Growth Curves, Helsinki, Finland). Assays were performed in triplicate. Relative growth rates were calculated by dividing the generation time of each DH10b transformed strain by the generation time of the wild-type DH10b strain which was included in each individual assay^78^. Growth rates between strains were compared using the Wilcoxon rank sum test with a Bonferroni adjustment for multiple comparisons. All statistical analysis were performed in R studio (version 1.0.143)^76^.

### Stability assays

*E. coli* DH10b transformants carrying different IncX3 plasmids were propagated in antibiotic-free LB medium at 37 °C with 180 rpm shaking for 10 days (∼180 generations) to determine their stability in an *E. coli* population. Cultures of each strain were daily diluted 1000-fold into 3 mL of fresh pre-warmed LB medium without antibiotics. On day 10, cultures were plated onto antibiotic-free LB agar and 100 randomly chosen colonies of each evolved line were replica-plated onto antibiotic-free and antibiotic-containing (1 mg/L of cefotaxime) LB agar plates. Plasmid presence was confirmed by colony PCR targeting the *taxC* gene of the IncX3 plasmids^2^. Colony growth on antibiotic-free but not on antibiotic-containing plates indicated the proportion of bacteria that lost the plasmid. Assays were performed in triplicate. The chance of *E. coli* DH10b keeping the plasmid was estimated for every plasmid using @Risk 6.3.1 (Palisade Corporation, Newfield, NY, USA), and the proportions of plasmid-harbouring colonies for each plasmid were compared.

### *Galleria mellonella* survival assays

*G. mellonella* caterpillars in the final-instar larval stage were obtained in bulk from Livefood UK Limited (Rooks Bridge, Somerset, United Kingdom) and stored at 15 °C in the dark on wood shavings prior to use. Ten randomly chosen larvae weighing between 250 mg and 350 mg were employed for each group of an experiment. Strains included in the assay were grown overnight in LB broth and washed twice in sterile phosphate-buffered saline (PBS). The optimal bacterial inoculum was determined by injecting 10 larvae with 10 μl of bacterial suspensions containing 10^4^ to 10^7^ CFU/larva of organism in PBS. Bacterial inoculum concentration was determined by viable bacterial count on LB agar identifying the inoculum which killed 50% of larvae after 24 hours incubation at 37 °C (LD_50_). The optimal inoculum was then injected into the hemocoels of the caterpillars via a left proleg using 25-μl Hamilton syringes (Cole-Parmer, London, United Kingdom). Following injection, larvae were incubated in petri dishes lined with filter paper at 37 °C for 96 h and scored for survival by 2 independent observers daily. Larvae were considered dead when they displayed no movement in response to touch. Two control groups were used per experiment, including larvae that were inoculated with PBS to control for any lethal effects of the injection process and larvae that received no injection. All *G. mellonella* survival assays were performed in triplicate using different batches of larvae. Survival curves were plotted using the Kaplan-Meier method and differences in survival were calculated by the log-rank test using R studio (version 1.0.143)^76^.

### Data availability

All data generated or analysed during this study are included in this published article (and its Supplementary Information files).

### Accession codes

The reported plasmid sequences are deposited in GenBank under the following accession numbers: KX618696 (pEC-NRS18), KX618697 (pEC-393), KX618698 (pEC-243) and KX618703 (pEC-125).
